# The Effectiveness of Mini Primer STR CODIS in DNA Degradation as the Effect of High-Temperature Exposure

**DOI:** 10.1155/2020/2417693

**Published:** 2020-12-23

**Authors:** Ahmad Yudianto, Fery Setiawan

**Affiliations:** ^1^Department of Forensic Medicine and Medicolegal, Faculty of Medicine, Universitas Airlangga, Indonesia; ^2^Forensics Study Program, School of Postgraduate, Universitas Airlangga, Indonesia; ^3^Human Genetics Study Group-Institute of Tropical Disease, Universitas Airlangga, Indonesia

## Abstract

**Background:**

More and more today, forensic identification through deoxyribonucleic acid (DNA) examination has achieved greater recognition in supporting Indonesia's law enforcement. Such examination is to determine the origin of a child, paternity cases, genealogical relation, or identifying unknown crime victims. However, along with the development of this DNA material examination, problems arise. DNA undergoes a degradation, commonly known as degraded DNA, which is one of the serious issues frequently encountered by forensic and DNA experts. Some forensic DNA experts take one of the alternatives to overcome this issue by implementing a mini primer set that is through a method to reduce the size of STR assays on DNA core locus examination.

**Methods:**

In this study, the writers conduct research using the mini primers of CSF1PO, FGA, and D21S11 of the molar teeth exposed to 500°C temperature for 20 and 30 minutes and 750°C for the same amount of time.

**Result:**

The findings show the DNA contents of molar teeth significantly (*p* < 0.05) decreased as the effect of high-temperature exposure. PCR result visualization shows CSF1PO is the only locus detected with mini primer exposed to 750°C temperature for 30 minutes (the highest exposure during this research).

**Conclusions:**

This finding suggests that this locus is potential in examining identification through DNA analysis, especially on a degraded condition as the effect of high-temperature exposure. Besides, this could accelerate the identification process especially on mass disaster events or criminal cases.

## 1. Introduction

More and more today, forensic identification through deoxyribonucleic acid (DNA) examination has achieved greater recognition in supporting Indonesia's law enforcement. Such examination is to determine the origin of a child, paternity cases, genealogical relation, or identifying unknown crime victims. This has been proven by the recognition of the examination invented by Sir Alec Jeffrey as one of the shreds of evidence in both judicial court and religion court since 1997. DNA identification played a significant role in identifying the victims of the Bali bombing back in 2002 [1, 2].

However, along with the development of DNA material examination, problems arise. One of the serious problems encountered by forensic DNA and other experts in this field is a DNA in degraded condition or commonly known as a degraded DNA [3, 4]. Some forensic DNA experts take one of the alternatives to overcome this issue by implementing a mini primer set that is through a method to reduce the size of Short Tandem Repeat (STR) assays on DNA core locus examination [5].

This research applied the average temperature of 500°C as treatment, the same as that in the previous research conducted by Thanakum (1999). Besides, the 750°C was applied, the same temperature as the research conducted by Sosiawan (2007) with 20 minutes duration, the longest duration of Thanakum's research, and 30 minutes duration, the longest duration of Sosiawan's [6, 7].

Nevertheless, until recently, there is no specific research on the effectiveness of the mini primer set to use as an alternative in forensic DNA identification using degraded DNA, especially on the DNA core. It is important to determine which loci are potential (especially CSF1PO, FGA, and D21S11) to apply in the degraded DNA examination.

The objective of this research is to determine the effectiveness rate of core DNA mini primer set utilization on CSF1PO, FGA, and D21S11 loci of assumed degraded DNA using the Polymerase Chain Reaction (PCR) method.

## 2. Materials and Method

This study is experimental laboratory research with a randomized posttest-only control group design. The samples of this study are 16 second-molar teeth of corpse T4. The variables of this study consist of FGA, CSF1PO, and D21S11 miniSTR CODIS (Short Tandem Repeat Combined DNA Index System) loci as dependent variables, and the independent ones are 500°C and 750°C temperature exposure for 20 and 30 minutes. The materials are DNAzol Reagent, ethanol 100%-70%, PCR mix, Nuclease Free Water, Agarose, Tris Boric Ethylene Diamine Tethraacetic Acid (EDTA) 0.5%, Marker 100 bp, and bromphenol blue 0.03%, mini-STRprimer CODIS:

mini CSF1PO (Promega Primer, Gen Bank Accession X14720)

5′-ACAGTAACTGCCTTCATAGATAG-3′

5′-GTGTCAGACCCTGTTCTAAGTA-3′

mini FGA (Promega primer, Gen Bank Accession M64982)

5′-AAATAAAATTAGGCATATTTACAAGC-3′

5′-GCTGAGTGATTTGTCTGTAATTG-3′

mini D21S11 (Promega primer, Gen Bank Accession AP000433)

5′-ATTCCCCAAGTGAATTGC-3′

5′-GGTAGATAGACTGGATAGACGA-3′

### 2.1. Data Collection Procedures

#### 2.1.1. Decalcifying the Second Molar Teeth

Molar teeth exposed to 500°C and 750°C temperature for 20-30' (minutes) were powdered using mortar. One gram of powdered teeth was put into an Eppendorf tube and decalcified with 40 ml of 0.5 M EDTA solution with pH 7.5. Afterward, it was vortexed and sonicated for 15 minutes and with centrifuge at 2000 rpm for 15 minutes. The process was monitored with saturated ammonium oxalate solution dripped into EDTA. The decalcification process stops when EDTA remains clear. The pellet was washed with 40 ml deionized H_2_O sterile and centrifuged at 2000 rpm for 15 minutes, and the supernatant was removed afterward. The washing procedure was repeated three times to ensure the sample was free from decalcification residue. This was the sample in which the DNA was going to be extracted.

#### 2.1.2. Extracting DNA from Blood and Sweat Smears Using DNAzol Reagent

The pellet obtained from the decalcification process was added with 1 ml DNAzol Reagent, vortexed, and incubated for 5 minutes at room temperature. The pellet was centrifuged at 10000 rpm for 10 minutes at 4°C. Viscous supernatant was moved to a new tube and added with 0.5 ml 100% ethanol (absolute). The tube was turned upside down, incubated at room temperature for 1-3 minutes, and centrifuged at 4000 rpm for 1-2 minutes at 4°C. The supernatant was carefully removed to prevent the removal of DNA (pellet). The pellet was washed twice with 0.8-1 ml of 75% ethanol. The tube was turned upside down 3-6 times each repetition. The tube was positioned upward for 0.5-1 minute, and the 75% ethanol was removed by pipetting or decanting. The pellet was dried up by opening the tube for 5-15 seconds. The pellet containing DNA was diluted on 25 *μ*l distilled water, sufficiently vortexed, and stored at -20°C temperature.

#### 2.1.3. Electrophoresis Using 2% Agarose Gel

Procedures in creating agarose gel were as follows: 40 ml TBE 0.5X mixed with 0.8 grams of agarose inside Erlenmeyer flask. The mixture was stirred for 15 minutes and put into an oven at 60-64°C until no agarose is adhering on the wall flask. The mixture was poured into electrophoresis molds and let the gel freeze. Afterward, TBE was poured evenly on the gel, and the comb was lifted carefully.

## 3. Results

The weight of the tooth samples (second molar teeth) taken from corpse T4 was measured before and after the treatment. The average weight of the tooth samples before and after the treatment are illustrated in Table 1.

Table 1 shows that the weight of the tooth samples decreases by 50-60% after the treatment. The following Table 2 indicates the average of DNA content from the tooth sample.

PCR Amplification: mini primer FGA, CSF1FO, and D21S11: [8, 9]

Table 2 shows the DNA content of the tooth sample decreases as the effect of high-temperature exposure. There are some uppercase numbers (1 until 6). Rather than functioning as a reference, these numbers indicate significance after a statistical analysis was conducted with the value of *p*, which is lower than 0.05. It suggests that the higher the temperature, the lower the DNA content of the tooth sample. The result of the Analysis of Variance (ANOVA) test indicates the effect of the treatment on DNA contents from the samples (significance rate 0.000 with significance limit *p* < 0.05). The result of the *t*-test indicates significant differences in DNA contents among different bone samples. It was after treatments (significance limit when*p* < 0.05), namely 500°C-20 minutes exposure: 500°C-30 minutes exposure; 500°C-20 minutes exposure:750°C–20 minutes exposure; 500°C-30 minutes exposure:750°C–30 minutes exposure; and 750°C-20 minutes exposure: 750°C–30 minutes.

.

### 3.1. The Effect of High-Temperature Exposure on FGA, CSF1PO, and D21S11 Loci of Tooth Sample DNA with Mini Primer Set

**Figure 1:**
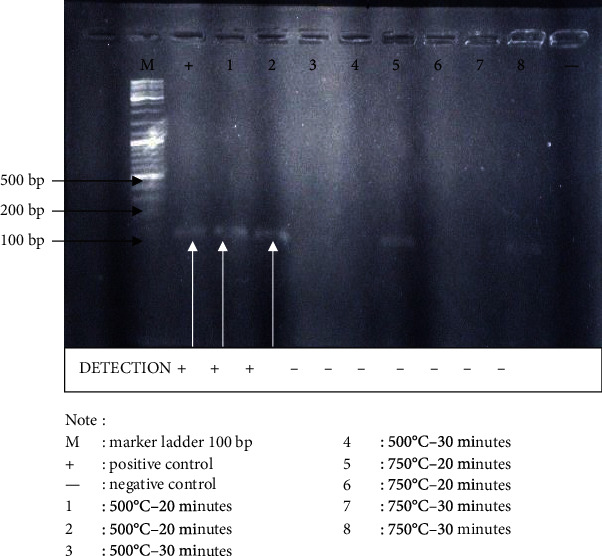
Visualization of FGA locus of the tooth sample PCR results using a mini primer.

The following are electrophoresis images with agarose gel 2% on Short Tandem Repeat Combined DNA Index System (STR CODIS) loci with the mini primer of Polymerase Chain Reaction (PCR) result after being exposed to 500°C for 20 and 30 minutes: exposed to 750°C for 20 and 30 minutes.  Figure 1 shows the electrophoresis visualization of the tooth sample FGA locus PCR results with agarose gel 2%.

The visualization of the tooth sample FGA locus PCR result with mini primer shows that the exposure of 500°C for 20 minutes is still detectable within 118-170 bp range, while the exposure of 500°C for 30 minutes and 750°C for 20 and 30 minutes is imperceptible.

The visualization of the PCR result presented in Figure 1 shows that only 500°C exposure for 20 minutes is detectable within the 118-170 bp range. The following image is the visualization of the tooth sample CSF1PO locus PCR result with agarose gel 2% after 500°C exposure for 20 and 30 minutes as well as 750°C exposure for 20 and 30 minutes.

**Figure 2:**
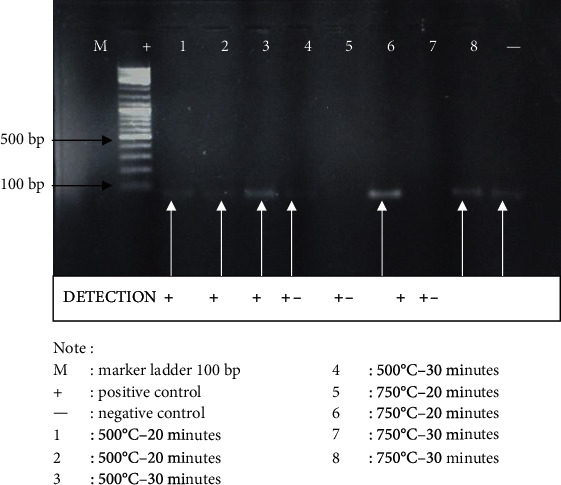
Visualization of CSF1PO locus of the tooth sample PCR results using a mini primer.

The visualization of the PCR result presented in Figure 2 shows that exposures of 500°C for 20 and 30 minutes, and 750°C for 20 and 30 minutes are detectable within the range of 89-129 bp. The following image is the visualization of the tooth sample D21S11 locus PCR result with agarose gel 2% after 500°C exposure for 20 and 30 minutes and 750°C for 20 and 30 minutes.

**Figure 3:**
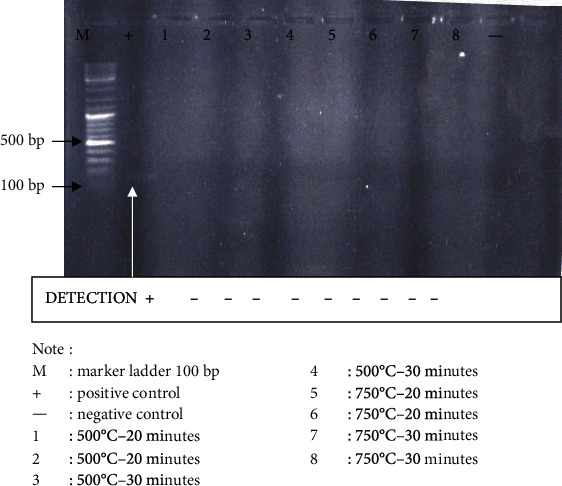
Visualization of D21S11 locus of tooth sample PCR results using a mini primer.

The visualization of the PCR result presented in Figure 3 illustrates the exposure of 500°C for 20 minutes until 750°C for 30 minutes within 153-221 bp range is undetectable.

The complete result of DNA examination detecting FGA, CSF1PO, and D21S11 loci on tooth samples using a mini primer after high-temperature exposure is presented in Table 3.

Table 3 shows that STR examination on tooth sample DNA through FGA locus with an exposure of 500°C for 20 minutes and 750°C for 20 minutes is still detectable (31.25% samples). Furthermore, CSF1PO locus on 500°C and 750°C for 20 and 30 minutes length of exposure is also detectable. However, DNA on the D21S11 locus is undetectable after 500°C for 20 minutes exposure until 750°C for 30 minutes exposure.

## 4. Discussion

DNA content is an important factor in forensic DNA examination that affects the success of DNA sample STR genotyping. The decreasing of DNA content by 1 ng potentially reduces the ability to detect Short Tandem Repeat (STR) up to 93% [10]. The minimum DNA content required in forensic DNA examination is 50 ng and 20 ng, respectively, while Butler (2005) argues that the minimum DNA content used in the STR examination is 0.5-2.5 ng [11, 12].

In addition to the DNA content of the sample, Polymerase Chain Reaction- (PCR-) based DNA examination also requires adequate DNA quality. DNA quality here means that the DNA must not be in a degraded condition. Severely degraded DNA may cause the primer used cannot adhere to the target DNA to be replicated [2, 13–15]. To obtain adequate visualization results, adequate DNA purity and proper DNA content are required so that the DNA can be used as a material in DNA examinations including the identification process and paternity test [15].

DNA degradation as the result of abnormal exposures, such as high temperatures, may be caused by irreversible damage on DNA hydrogen bonds. This condition causes damage to DNA's purine-pyrimidine coupling as the main component of the DNA structure [4, 16].

This study employed samples taken from a corpse with unknown residency (*Tempat Tinggal Tidak Tetap/T4*). The degradation of DNA samples after death is an endogenous process beginning soon after death. DNA degradation may occur altogether with the decomposition process through autolysis and bacterial decomposition.

Postmortem DNA degradation as the result of the autolysis process can occur in the forms of pyrimidine modification, baseless sites, intermolecular crosslink, and DNA's low molecular weight as the result of strand breakage [4, 15, 16].

The findings of this study show that only the CSF1PO locus with the mini primer is detectable after being exposed to 750°C temperature for 30 minutes, the highest temperature and the longest duration applied in this study. This finding indicates that DNA examination on tooth samples through STR locus detection results in different responses on different temperatures exposed to tooth samples.

Teeth are also composed of the most complete mineral hard tissue. The mineral is known as apatite, which mostly comprises hydroxyapatite. Teeth also contain essential secondary minerals, which are higher compared to those of bones, namely calcite, limonite, pyrite, and vivianite resulting in teeth with strong endurance or protection [14, 17].

The use of a mini primer is an alternative to substitute standard primer on DNA with the degraded condition. The use of standard primer on degraded DNA will be less successful. Mini primer is a result of standard primer redesigning through amplicon size reduction by shifting the position of primer as close as possible to the loop area [15]. Mini primer is an interesting alternative in conducting DNA forensic analysis on degraded DNA, compared to forensic analysis using mtDNA.

The success in detecting this locus is reinforced by the different amplicon product and guanine-cytosine coupling or GC content on each locus. GC content is with higher stability against denaturation compared to adenine-thymine coupling [15].

The measurement of the GC content ratio shows a significant result. The result of the GC ratio measurement of each locus is CSF1PO: 42.6%; FGA: 35.7%; and D21S11: 34.1%. Furthermore, consecutive adenine is a potential target of DNA degradation caused by high temperatures. Adenine is the easiest base to oxidize [15, 16].

The hurdle/obstacle in this research is the obtained sample is not homogenous because of the limitation in the number of the corpse with unknown residency. Another problem is the length of time to order the primer in advance.

## 5. Conclusion

CSF1PO locus, still detected with a mini primer on 750°C temperature exposure for 30 minutes, is potential in examining identification through DNA analysis. This is true especially for degraded condition as the effect of being exposed to high temperature, accelerating the identification process, mainly that of mass disasters and other criminal cases as well. The limitation of this study is the use of the locus DNA is only limited to the population in Java so that we cannot generalize the suggestion in DNA examining procedures. Further study can examine each population in Indonesia so that we can generalize and promote the use of DNA even though in a high exposure temperature. This study correlates to the other study especially in the DNA examination after exposure to high temperature.

## Figures and Tables

**Table 1:** Average weights of the tooth samples before and after the treatment (high-temperature exposure).

Average weights of tooth sample (grams)			
Before treatment	After treatment		
2.8	500°C	20'	2.0
2.4		30'	1.4
2.7	750°C	20'	1.7
2.5		30'	1.3

**Table 2:** Average DNA content of the tooth sample.

Exposure (°C)		Tooth DNA content (*x* ± SD) ng/*μ*l
Without exposure		269.25 ± 10.25
500°C	20'	147.32 ± 9.07^1,2,3^
	30'	128.40 ± 5.41^1,4,5^
750°C	20'	110.37 ± 9.51^2,4,6^
	30'	72.18 ± 3.09^3,5,6^

Note: *X*: Average DNA content; SD: standard deviation.

**Table 3:** Results of DNA detection on STR CODIS examining the effect of high-temperature exposure on tooth sample DNA with various temperature treatment and length of treatment on FGA, CSF1PO, and D21S11 loci.

Exposure		FGA		CSF1PO		D21S11		Total (% + result)
		Detection		Detection		Detection		
		+	−	+	−	+	−	
500°C	20'	2	2	4	0	0	4	50%
	30'	2	2	2	2	0	4	50%
750°C	20'	1	3	2	2	0	4	25%
	30'	0	4	1	3	0	4	8.5%
Total + DNA after exposure (%)		5/16 31.25%		9/16 (56.25%)		0%		

## Data Availability

The (table data and picture) data used to support the findings of this study are included in the article. The author and the co-author strongly agree to share it and included it in the main manuscript.
